# Circular RNA circ_0000423 promotes gastric cancer cell proliferation, migration and invasion via the microR-582-3p/Disheveled-Axin domain containing 1 axis

**DOI:** 10.1080/21655979.2021.1997696

**Published:** 2021-12-21

**Authors:** Chao-Qun Huang, Ping Yang, Jiuyang Liu, Yin-Ping Wang, Dan-Dan Hao, Xiaojun Yang

**Affiliations:** a Department of Gastrointestinal Surgery, Zhongnan Hospital of Wuhan University, Wuhan, China; b Hubei Cancer Clinical Study Center & Hubei Key Laboratory of Tumor Biological Behaviors, Wuhan, China; c Department of GastroenterologyThe Clinical Medical Research Center of Peritoneal Cancer of Wuhan, Wuhan, China; d Department of Gastroenterology, Zhengzhou Central Hospital, Zhengzhou, Henan, China

**Keywords:** Gastric cancer, circ_0000423, MiR-582-3p, DIXDC1, competitive endogenous RNA, bioinformatics

## Abstract

For humans, gastric cancer (GC) is a common malignancy. Multiple circular RNAs (circRNAs) have been confirmed to be important cancer-promoting or tumor-suppressive factors. The present study discusses the roles and mechanisms of circ_0000423 in GC development. In this study, circ_0000423 expression in GC patient tissue samples and cell lines was detected via quantitative real-time polymerase chain reaction. Disheveled-Axin domain containing 1 (DIXDC1) expression in GC cells was examined via Western blot. Besides, cell counting kit-8 was utilized for detecting GC cell viability. GC cell migration and invasion were examined through Transwell assays. Bioinformatics and dual-luciferase reporter gene assays were employed to verify the regulatory relationships between microRNA-582-3p (miR-582-3p) and circ_0000423 or DIXDC1. In the present study, we demonstrated that circ_0000423 was highly expressed in GC. Circ_0000423 knockdown suppressed GC cell viability, migration and invasion. Moreover, miR-582-3p was confirmed as a direct target of circ_0000423, and an upstream regulator of DIXDC1. MiR-582-3p inhibition or DIXDC1 overexpression could reverse the above-mentioned effects of knocking down circ_0000423 on GC cells. In conclusion, circ_0000423 facilitates GC progression by modulating the miR-582-3p/DIXDC1 axis.

## 1. Introduction

Gastric cancer (GC), as the fifth most common carcinoma worldwide, ranks third among the causes of cancer-related death [1]. It is estimated that more than 1 million patients are diagnosed with GC every year, and half of them are from China [2,3]. GC is associated with multiple pathological factors, and both environmental and genetic factors have a role in its etiology; despite dramatic advances in surgery and adjuvant therapy for GC, since over 80% of GC patients are diagnosed with advanced cancer, the mortality of GC still is very high: the 5-year overall survival rate is less than 30% due to frequent tumor recurrence and metastasis [4–6]. In this context, seeking new diagnostic biomarkers and therapy targets for GC becomes urgent.

Non-coding RNA (ncRNA) has become a hot spot in cancer research. As a newly discovered class of ncRNA, circular RNAs (circRNAs) are widely expressed in eukaryotes; they are formed by non-canonical splicing and have a covalently closed loop with neither 3ʹ poly(A) tail nor 5ʹ end [7,8]. The special structure of circRNAs makes it relatively resistant to degradation mediated by exonuclease [8,9]. CircRNAs are strongly linked to the development of various diseases, especially cancer [10]. For instance, circ_0007294 expression is markedly increased in triple-negative breast carcinoma, and its high expression is strongly associated with lymph node metastasis and advanced clinical stage [11]; circ_0101432 inhibits hepatocellular cancer cell apoptosis through targeting miR-622 and miR-1258, and up-regulating mitogen-activated protein kinase 1 expression [12]. In the present study, bioinformatics analysis suggested that circ_0000423 was highly expressed in GC tissues. Circ_0000423 is formed by reverse splicing of protein phosphatase 1 regulatory subunit 12A (PPP1R12A) mRNA. Reportedly, circ_0000423 expression is up-regulated in colon carcinoma tissues, and high circ_0000423 expression is strongly related to the short survival time of patients [13]. Nonetheless, the specific role of circ_0000423 in GC is still not fully elucidated, and further exploration is needed.

We hypothesized that circ_0000423 played an important role in GC progression. The current study was aimed at exploring the role and mechanism of circ_0000423 in GC, providing a theoretical basis for clarifying the mechanism of the progression of this deadly disease.

## 2. Materials and methods

### 2.1. Clinical samples and ethics statement

A total of 43 GC patients (38–72 years old, 28 males and 15 females) who received gastrectomy at the First Affiliated Hospital of Zhengzhou University from 2016 to 2019 were enrolled in the study. Based on tumor, lymph node metastasis and tumor node metastasis (TNM) grading according to the International Union Against Cancer (7th edition), there were 14 cases at stage I, 10 cases at stage II, 19 cases at stage III. The tumorous and adjacent non-tumorous tissues (5 cm away from the tumor) were surgically removed and frozen with liquid nitrogen and then kept at −80°C. None of the participants had undergone chemotherapy or radiotherapy before tissue collection. The study was endorsed by the Ethics Committee of First Affiliated Hospital of Zhengzhou University (approval no. 201606032), and we obtained the written informed consent of each patient. The present study was carried out following the *Declaration of Helsinki*.

### 2.2. Cell culture

From the Cancer Institute and Hospital of the Chinese Academy of Medical Sciences (Beijing, China) and the American Type Culture Collection (Manassas, VA, USA), a human renal epithelial cell line (293 T), 4 kinds of GC cell lines (HGC-27, AGS, MGC-803 and MKN-45) and a human gastric epithelial cell line (GES-1). These cells were cultured in Dulbecco’s modified Eagle’s medium (DMEM; Gibco, Grand Island, NY, USA) containing 100 U/ml penicillin, 0.1 mg/ml streptomycin and 10% fetal bovine serum (FBS, Gibco, Grand Island, NY, USA) in 5% CO_2_ at 37°C.

### 2.3. CircRNA expression profile analysis

From the Gene Expression Omnibus (GEO) (http://www.ncbi.nlm.nih.gov/geo) database, we downloaded three datasets (GSE78092, GSE89143, and GSE141977) with GC circRNA expression profiles. To screen out differentially expressed circRNAs, the GEO2R online software was utilized to analyze them.

### 2.4. Cell transfection

From GenePharma (Shanghai, China), miR-582-3p inhibitors (anti-miR-582-3p: 5ʹ-GGUUCAGUUGUUCAACCAGUUA-3ʹ) and miR-582-3p mimics (5ʹ-UAACUGGUUGAACAACUGAACC-3ʹ), their negative controls (miR-con mimics: 5ʹ- UACGACUGAUACGAUCAUCAGA-3ʹ, and anti-miR-con: 5ʹ-GAAUGACUCACUGCUGAUUGUU-3ʹ), small interfering RNAs (siRNAs) targeting circ_0000423 (si-circ_0000423#1:5ʹ-ACCCAGCCCTGTAAGACCAGT-3ʹ and circ_0000423#2: 5ʹ-AAAAGGCCACCCAGCCCTGTA-3ʹ) and siRNA control (si-NC) were obtained. To construct overexpression vectors of Disheveled-Axin domain containing 1 (DIXDC1) (pcDNA3.1-DIXDC1) and circ_0000423 (pCD5-circ_0000423), the full-length cDNA of DIXDC1 sequence was amplified and cloned into the pcDNA3.1 vector (GenePharma, Shanghai, China); the full-length cDNA of circ_0000423 was amplified and inserted into the pCD5-ciR vector (Greenseed Biotech, Guangzhou, China), which contained a front and back circular frame to promote RNA circularization. They were transfected into AGS and MGC-803 cells employing Lipofectamine® 2000 (Invitrogen, Carlsbad, CA, USA) according to the manufacturer’s instructions.

### 2.5. Quantitative real-time polymerase chain reaction (qRT-PCR) and RNase R treatment

A TRIzol kit (Invitrogen, Carlsbad, CA, USA) was utilized for extracting the total RNA, and the PrimeScript™ RT Reagent Kit with gDNA Eraser (Takara Bio Inc., Shiga, Japan) was employed to reversely transcribe RNA into complementary DNA (cDNA). qRT-PCR was implemented with a SYBR® Premix Ex Taq™ II kit (Takara Bio Inc., Shiga, Japan) in Applied Biosystems 7500 Real-time PCR System (Applied Biosystems, Inc. Carlsbad, CA, USA). GAPDH and U6 were used as the internal reference for genes and miRNAs expression, respectively. For RNase R treatment, 1 μg of RNA was incubated with 1 U/μg RNase R (Epicenter, Madison, Wisconsin, USA) for 20 min at 37°C, and then reverse transcription and PCR was performed. Primer sequences are shown in Table 1.

**Table 1.** Primer sequences used in this study

Gene	Sequence
circ_0000423	F: 5ʹ-ACAGCAGCAGGCTAGAAAAG-3ʹ R: 5ʹ-TGTCCTAAGCAGGAAAAACA-3’
miR-582-3p	F: 5ʹ-GCACACATTGAAGAGGACAGAC-3ʹ R: 5ʹ-TATTGAAGGGGGTTCTGGTG-3’
DIXDC1	F: 5ʹ-TGCATGTTATGGAGACGCAGAAG-3ʹ R: 5ʹ-AGGTGCTGCTGACAGTTGGAGA-3’
RREB1	F: 5ʹ-GGGCTTATCCCCCAGTCAAA-3ʹ R: 5ʹ-TCTCCGCATCCGACTGACT-3’
INO80D	F: 5ʹ-CCTGATGACTTACAAGATTTTGATTT-3ʹ R: 5ʹ-CTCCTCAGCCTCTTCGGTAG-3’
U6	F: 5ʹ-CTCGCTTCGGCAGCACA-3ʹ R: 5ʹ-AACGCTTCACGAATTTGCGT-3’
GAPDH	F: 5ʹ-TGGTCACCAGGGCTGCTT-3ʹ R: 5ʹ-AGCTTCCCGTTCTCAGCC-3’

F, forward; R, reverse.

### 2.6. Cell counting kit-8 (CCK-8) assay

After the transfection with the plasmids or oligonucleotides, MGC-803 and AGS cells (2 × 10^3^ cells/well in a 96-well plate) were cultured for 0, 24, 48, and 72 h. Next, each well was added with CCK-8 reagent (10 μL; Dojindo, Tokyo, Japan), followed by the incubation for 2 h. After that, the absorbance of the cells in each well at 450 nm was detected with a microplate reader (Bio-Rad, Hercules, CA, USA) [14].

### 2.7. Transwell assays

Using the Transwell chamber (24-well; Corning Incorporated, Corning, NY, USA), cell migration and invasion were measured via Transwell assays [15]. The processes of migration assay and invasion assay were similar. For invasion assay, however, the filter of upper compartment was pre-coated with Matrigel (BD Bioscience, San Jose, CA, USA). GC cells (1 × 10^4^ cells/well) cultured in FBS-free medium were transferred to the top compartment, and 10% FBS-containing DMEM was injected into the lower compartment. The cells were cultured for 24 h, and the cells on the top membrane surface were removed using cotton swabs, and the cells on the lower surface were fixed with methanol and stained with crystal violet (0.25%; Sigma, St. Louis, MO, USA). In three random fields under the microscope, the numbers of migrated or invaded cells were analyzed.

### 2.8. Luciferase reporter gene assay

The circ_0000423/DIXDC1 mRNA 3ʹUTR sequence containing binding sites to miR-582-3p were amplified and cloned into the pGL3 Basic reporter vector (Promega, Madison, WI, USA) to establish wild type (WT) circ_0000423-WT/DIXDC1-WT reporter vectors. Besides, the mutant type (MUT) circ_0000423/DIXDC1 mRNA 3ʹUTR sequence was inserted into pGL3 Basic reporter vectors to obtain the circ_0000423-MUT/DIXDC1-MUT1/2/1&2 reporter vector. Circ_0000423-WT/DIXDC1-WT or circ_0000423-MUT/DIXDC1-MUT1/2/1&2 and miR-582-3p mimics or miR-con mimics were co-transfected into 293 T cells, respectively. Ultimately, a Dual-Luciferase Assay System (Promega, Madison, WI, USA) was employed for determining the relative luciferase activity of the cells in each group.

### 2.9. Western blot

To obtain the total protein, protease inhibitors-containing radioimmunoprecipitation assay (RIPA) lysis buffer was applied to lyse cells. After being separated by sodium dodecyl sulfate polyacrylamide gel electrophoresis (SDS-PAGE), the protein was transferred to a polyvinylidene difluoride (PVDF) membrane (Amersham Bioscience, Piscataway, NJ). Subsequently, the membrane was blocked in 5% skim milk and then incubated with primary antibodies for DIXDC1 (ab226210, Abcam, Cambridge, MA, USA) and GAPDH (ab181602, Abcam, Cambridge, MA, USA) overnight at 4°C. After that, the membranes were incubated with secondary antibodies for 1 h at room temperature. A chemiluminescence detection system (Beyotime Biotechnology, Shanghai, China) was adopted to develop the protein bands.

### 2.10. Immunohistochemistry (IHC)

GC tissue and adjacent normal tissue were fixed in 4% paraformaldehyde for 24 h and embedded in paraffin. Serial sections of 4-μm thick were used to conducted IHC assay [16]. Briefly, the sections were dewaxed, hydrated, and incubated with primary antibody at 4°C overnight in a wet box. The next day, the sections were incubated with secondary antibodies for 2 h. Then, the diaminobenzidine (DAB) system was used to develop the color, and the sections were counterstained with hematoxylin. Finally, the stained section images were captured using a microscope.

### 2.11. Statistical analysis

All experiments were conducted in triplicate. The tool for statistical analysis was GraphPad Prism 8.0 (GraphPad Software, Inc., San Diego, CA, USA). All data were represented by the ‘mean ± standard deviation’. Independent-sample *t*-test was conducted to compare the measurement data between the two groups. One-way analysis of variance (ANOVA) with Tukey’s post-hoc test was conducted to compare the data among more groups. Correlation analysis was conducted through Pearson’s correlation coefficient analysis. A difference was of statistical significance when *P* < 0.05.

## 3. Results

In this study, we investigated the expression pattern, function and molecular mechanism of circ_0000423 in GC progression through bioinformatics, function-gain and function-loss experiments. It was revealed that, circ_0000423, which is highly expressed in GC, promotes the GC cell proliferation, migration and invasion via regulating microR-582-3p (miR-582-3p)/Disheveled-Axin domain containing 1 (DIXDC1) axis.

### 3.1. Circ_0000423 is high-expressed in GC tissues and cell lines

**Figure 1.**
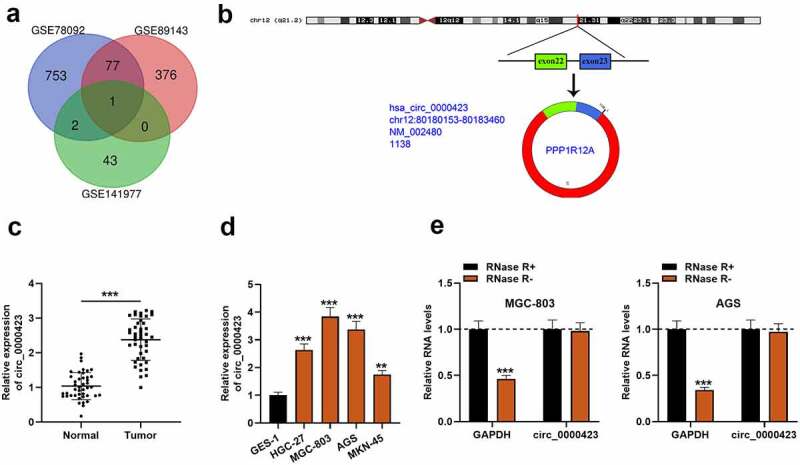
Circ_0000423 expression is significantly up-regulated in GC tissues and cell lines

First of all, the microarray data of GSE78092, GSE89143 and GSE141977 downloaded from the GEO database were analyzed, and in each dataset, there were multiple up-regulated circRNAs. However, circ_0000423, was the only circRNA which was up-regulated in GC tissues in all of the three GEO datasets (Figure 1a). Hence, circ_0000423 was selected for further research. Circ_0000423 is formed by head-to-tail splicing of PPP1R12A transcript (Figure 1b). Then, qRT-PCR was employed to examine circ_0000423 expression in 43 pairs of GC and normal para-tumorous tissues, and it was found that circ_0000423 expression was elevated in GC tissues as against para-cancerous tissues (Figure 1c). Additionally, the amplification products of PCR were evaluated by Sanger sequencing, and the result validated the circular structure of circ_0000423 (Figure S1) . In comparison to GES-1 cells, circ_0000423 expression was markedly up-regulated in HGC-27, MGC-803, AGS and KN-45 cells (Figure 1d). Moreover, the stability of circ_0000423 in AGS and MGC-803 cells was further verified using RNase R exonuclease. RNase R treatment could degrade GAPDH, yet it did not affect circ_0000423 (Figure 1e). These findings suggested that up-regulated circ_0000423 expression might be implicated in GC development.

(a) Venn diagram was used to show the differentially expressed circRNAs in GSE78092, GSE89143 and GSE141977. (b) The schematic diagram of circ_0000423, which was generated from PPP1R12A transcript. (c) Circ_0000423 expression in 43 pairs of GC tissues and para-tumorous tissues was detected by qRT-PCR. (d) Circ_0000423 expression in GES-1 cells and 4 GC cell lines (AGS, MGC-803, HGC-27 and MKN-45) was detected by qRT-PCR. (e) The effect of RNase R on circ_0000423 was evaluated by qRT-PCR, to show the stability of circ_0000423. **P < 0.01 and ***P < 0.001.

### 3.2. Circ_0000423 knockdown inhibits AGS and MGC-803 cell multiplication, migration and invasion

**Figure 2.**
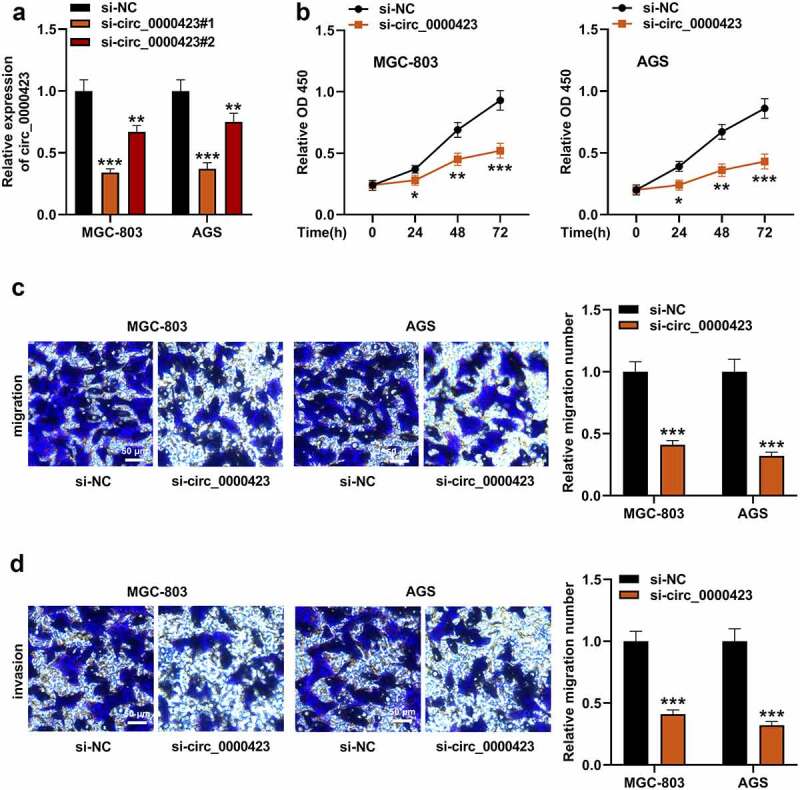
Circ_0000423 knockdown suppresses the proliferation, migration and invasion of MGC-803 and AGS cells

To ascertain circ_0000423’s role in GC development, AGS and MGC-803 cells with higher circ_0000423 expression, were transfected with si-circ_0000423#1 and si-circ_0000423#2 as well as si-NC to construct cell models with low circ_0000423 expression. Knockdown efficiency of si-circ_0000423#1 was higher, so it was used in subsequent experiments (Figure2a). Subsequently, we investigated the impacts that circ_0000423 had on GC cell growth, migration and invasion. CCK-8 assay showed that knocking down circ_0000423 inhibited AGS and MGC-803 cell viability (Figure2b). Transwell analysis suggested that circ_0000423 knockdown significantly inhibited the migration and invasion of MGC-803 and AGS cells (Figure2c-d).

(a) AGS and MGC-803 cells were transfected with si-circ_0000423#1, si-circ_0000423#2 or si-NC, and qRT-PCR was conducted for detecting circ_0000423 expression. (b) The impacts that knocking down circ_0000423 had on the growth of GC cells were detected by CCK-8 assay. (c) The impacts of knocking down circ_0000423 had on the migration of GC cells were detected by Transwell assay. (d) The impacts of knocking down circ_0000423 had on the invasion of GC cells were detected by Transwell assay. *P < 0.05, **P < 0.01 and ***P < 0.001.

### 3.3. MiR-582-3p is the direct downstream target of circ_0000423

**Figure 3.**
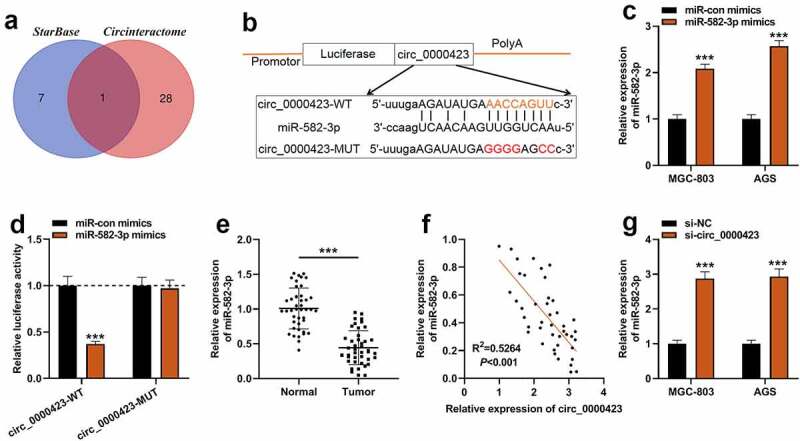
Circ_0000423 directly targets miR-582-3p

To dig deeper into the downstream mechanism of circ_0000423, bioinformatics tools were applied to predict the potential target miRNAs of circ_0000423. It was predicted that miR-582-3p was the only target of circ_0000423 predicted by both CircInteractome (https://circinteractome.nia.nih.gov/) and StarBase (https://starbase.sysu.edu.cn/) (Figure 3a, b) [17,18]. qRT-PCR showed that AGS and MGC-803 cells were successfully transfected with miR-582-3p mimics (Figure 3c). Dual-luciferase reporter gene assay indicated that miR-582-3p could dramatically reduce circ_0000423-WT’s luciferase activity yet had no significant impact on circ_0000423-MUT’s luciferase activity, suggesting that circ_0000423 could directly bind to miR-582-3p (Figure 3d). Subsequently, qRT-PCR showed that miR-582-3p expression was markedly elevated in GC tissues as opposed to para-cancerous tissues (Figure 3e). Meanwhile, Pearson correlation analysis indicated that circ_0000423 expressions and miR-582-3p expressions were inversely correlated in GC samples (figure 3f). Furthermore, in GC cells, knockdown of circ_0000423 enhanced miR-582-3p expression (Figure 3g).

(a) Venn diagram of the downstream miRNA targets predicted by CircInteractome (https://circinteractome.nia.nih.gov/) and StarBase (http://starbase.sysu.edu.cn/). (b) The binding site of circ_0000423 and miR-582-3p was predicted by StarBase. (c) The efficiency of miR-582-3p overexpression in MGC-803 and AGS cells was detected by qRT-PCR. (d) The luciferase activity of the cells co-transfected with miR-582-3p mimics and circ_0000423-MUT reporter or circ_0000423-WT reporter was detected. (e) MiR-582-3p expression in GC tissues and para-cancerous tissues was detected by qRT-PCR. (f) Pearson correlation analysis was used to evaluate the correlation between miR-582-3p expression and circ_0000423 expression in the 43 cases of GC tissues. (g) The expression of miR-582-3p in AGS cells and MGC-803 cells after knocking down circ_0000423 was detected by qRT-PCR. ***P < 0.001.

### 3.4. The circ_0000423/miR-582-3p axis partakes in modulating the biological behaviors of GC cells

**Figure 4.**
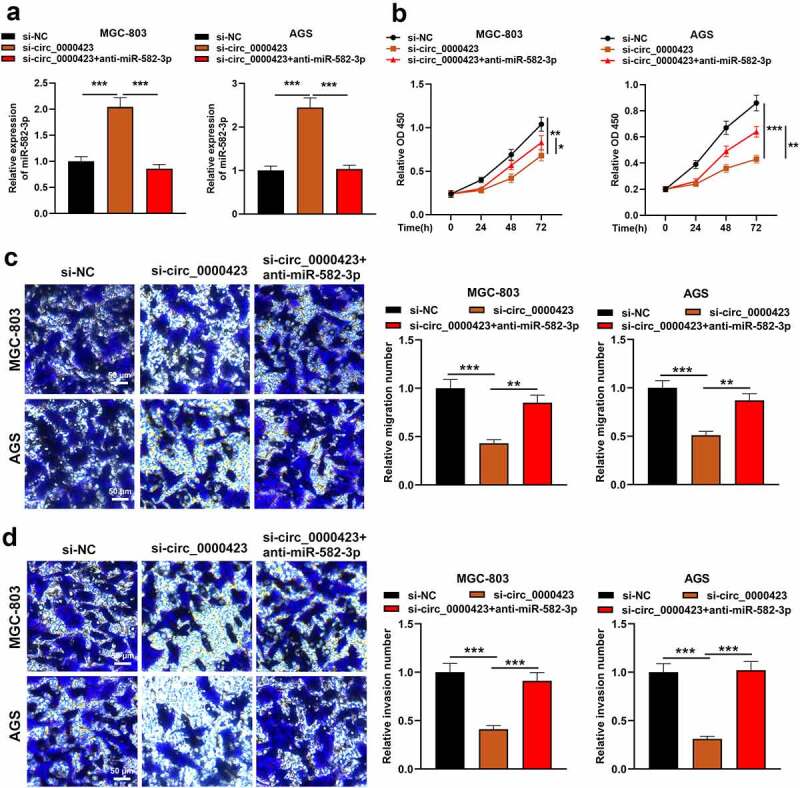
MiR-582-3p inhibition weakens the inhibitory effects of silencing circ_0000423 expression on the malignant biological behaviors of GC cells

To pinpoint the role of circ_0000423/miR-582-3p axis in GC development, MGC-803 and AGS cells were transfected with si-NC, si-circ_0000423 or si-circ_0000423 + miR-582-3p inhibitor (Figure 4a). It was revealed miR-582-3p inhibition attenuated the inhibiting effects of knocking down circ_0000423 on the growth, migration and invasion of MGC-803 and AGS cells (Figure 4b-d). These data suggested that the circ_0000423/miR-582-3p axis participated in modulating the biological behaviors of GC cells.

(a) AGS and MGC-803 cells were transfected with si-NC, si-circ_0000423 or si-circ_0000423 + miR-582-3p inhibitor, and qRT-PCR was conducted for detecting miR-582-3p expression. (b) The growth of MGC-803 cells and AGS cells was detected by CCK-8 assay. (c) The migration of MGC-803 cells and AGS cells was detected by Transwell assay. (d) The invasion of MGC-803 cells and AGS cells was detected performing Transwell assay.*P < 0.05, **P < 0.01 and ***P < 0.001.

### 3.5. MiR-582-3p can target DIXDC1

**Figure 5.**
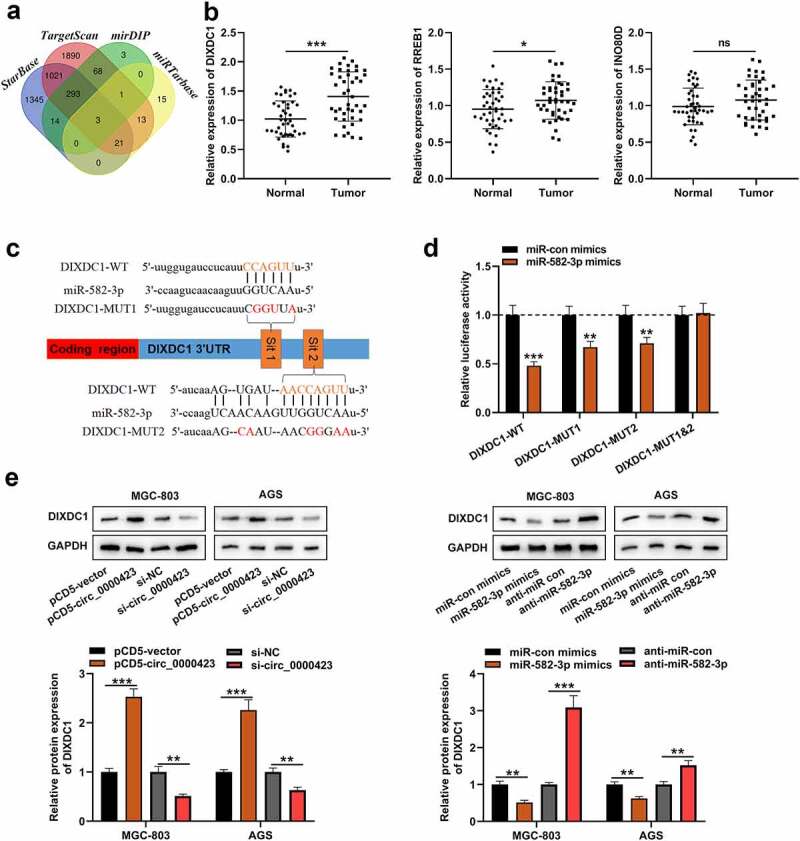
MiR-582-3p targets DIXDC1

Subsequently, the downstream mRNAs of miR-582-3p were predicted, and three of them (DIXDC1, RREB1 and INO80D) were predicted by all of the four tools, StarBase (https://starbase.sysu.edu.cn/), TargetScan (http://www.targetscan.org/vert_72/), mirDIP (http://ophid.utoronto.ca/mirDIP/index.jsp) and miRTarbase (http://mirtarbase.mbc.nctu.edu.tw/php/index.php) databases (Figure 5a). qRT-PCR and IHC showed that the expression of DIXDC1 was significantly highly expressed in the GC tissues (Figure 5b and Figure S2), while the changes of RREB1 expression and INO80D expression in GC tissues, compared with those in adjacent tissues, were not that significant (Figure 5b). The binding sites between DIXDC1 mRNA 3ʹUTR and miR-582-3p were presented by bioinformatics (Figure 5c). Subsequently, dual-luciferase reporter gene assay showed that miR-582-3p could reduce the luciferase activity of DIXDC1-WT reporter and DIXDC1-MUT1/2 reporters yet fail to significantly influence the luciferase activity of DIXDC1-MUT1&2 reporter (Figure 5d). Western blot assay showed that knocking down circ_0000423 or up-regulating miR-582-3p expression decreased DIXDC1 expression in GC cells, while circ_0000423 overexpression and miR-582-3p inhibition promoted DIXDC1 expression (Figure 5e). These results suggested that DIXDC1 was target gene of miR-582-3p, and could be positively regulated by circ_0000423.

(a) Venn diagram of the downstream targets of miR-582-3p predicted by StarBase, mirDIP, miRTarbase and TargetScan. (b) The expressions of DIXDC1, RREB1 and INO80D mRNA in GC tissues and matched adjacent tissues were detected through qRT-PCR. (c) The binding sites between DIXDC1 mRNA 3ʹUTR and miR-582-3p were predicted by StarBase. (d) The luciferase activity of cells co-transfected with miR-582-3p mimics and DIXDC1-WT reporter or DIXDC1-MUT1/2/1&2 reporter was measured by dual-luciferase reporter gene assay. (e) Western blot was applied to detect the effects of circ_0000423 and miR-582-3p on DIXDC1 expression.NS: differences were not statistically significant; *P < 0.05, **P < 0.01 and ***P < 0.001.

## 4. Discussion

GC is one of the most common malignancies of the digestive system; due to its insidious early symptom, high aggressiveness, and resistance to chemotherapy and radiotherapy, GC has a relatively high mortality [19]. Therefore, exploring the biological mechanism underlying GC occurrence and development and identifying more biomarkers will help to develop better treatment strategies.

CircRNAs are vital in the tumorigenesis and progression of multiple cancers. For instance, in GC tissues, some circRNAs are differentially expressed and partake in regulating the malignancy of cancer cells [20]. For example, circ_006100 expression is enhanced in GC tissues, and its high expression is closely associated with poor differentiation of tumor tissues, high TNM stage and lymph node metastasis [21]. Circ-RPL15 regulates miR-502-3p/OLFM4/STAT3 axis to regulate the malignant phenotype of GC cells, thus facilitating GC progression; abnormal circ-RPL15 expression can predict the GC patient’s poor prognosis [22]. Circ_0004872 expression is remarkably down-regulated in GC tissues, and circ_0004872 overexpression inhibits GC cell growth and metastasis *in vitro* and *in vivo* [23]. Circ_0001546 modulates ATM/Chk2/p53 pathway via repressing miR-421, thereby inhibiting GC cell proliferation and resistance to oxaliplatin [24]. Our study found that circ_0000423 was highly expressed in GC tissues and cells, and circ_0000423 knockdown restrained GC cell proliferation, migration and invasion. To the best of our knowledge, this is the first work to investigate the role of circ_0000423 in GC.

Emerging evidence shows that circRNA has multiple binding sites for miRNAs and may act as a miRNA sponge, thereby modulating the translation process of mRNA [25,26]. This competitive endogenous RNA (ceRNA) mechanism is reported to participate in regulating the pathogenesis of many human diseases, including GC [27,28]. MiR-582-3p has tumor-suppressive properties in several human malignancies. For example, it represses the growth and cell cycle progression of acute myeloid leukemia cells via inhibiting cyclin B2 [29]. MiR-582-3p suppresses the malignancy of prostate cancer cells through regulating TGF-β pathway [30]. Reportedly, in GC, miR-582-3p is low-expressed, and the transfection of miR-582-3p mimics inhibits GC cell growth, migration and invasion [31], which is in line with the findings of our study. Moreover, herein, it was confirmed that circ_0000423 directly targeted miR-582-3p to repress its expression, which helps better understand the mechanism of miR-582-3p dysregulation in GC.

Disheveled-Axin domain containing 1 (DIXDC1) is a new type of Disheveled-Axin domain protein [32]. There is growing evidence suggesting that DIXDC1 has carcinogenic effects in many cancers, for example, prostate cancer, glioma, non-small cell lung carcinoma and acute myeloid leukemia [33–37]. Reportedly, DIXDC1 is high-expressed in GC and is linked to advanced TNM stage, lymph node metastasis and poor prognosis [38]. By up-regulating MMPs expression, down-regulating E-cadherin expression and enhancing the nuclear accumulation of β-catenin to activate the WNT signaling, DIXDC1 participates in promoting GC cell invasion and metastasis [38,39]. Reportedly, DIXDC1 is a target gene of miR-154 in GC cells [40]. In this work, DIXDC1 was also identified as a target gene of miR-582-3p. Additionally, it was revealed that circ_0000423 promoted DIXDC1 expression in GC cells. These findings suggest that circ_0000423 regulates DIXDC1 expression via serving as a ceRNA of miR-582-3p in GC cells.

Many circRNAs have been used as diagnostic markers for cancers to predict the prognosis of the patients [41,42]. Due to the limitation of the sample number in the present work, the potential of circ_0000423 as a prognostic biomarker for GC has not been evaluated. In the following work, more patients should be recruited to solve these issues. In addition, the biological function of circ_0000423/miR-582-3p/DIXDC1 axis in GC development needs to be further validated with *in-vivo* models in the following studies.

## 5. Conclusion

To sum up, our study reports that circ_0000423 expression is enhanced in GC, and circ_0000423 can promote GC cell proliferation, migration and invasion through modulating miR-582-3p/DIXDC1 axis. These findings provide a novel explanation of GC progression.

## Supplementary Material

Supplemental Material

## Data Availability

The data used to support the findings of this study are available from the corresponding author upon request.

## Highlight

- Circ_0000423 expression is up-regulated in gastric cancer tissues and cells.
- Circ_0000423 promotes gastric cancer cell viability, migration and invasion.
- Circ_0000423 acts as a competing endogenous RNA (ceRNA) of miR-582-3p to regulate DIXDC1 expression.
