# Supplementary material for: Circular RNA circ_0000423 promotes gastric cancer cell proliferation, migration and invasion via the microR-582-3p/Disheveled-Axin domain containing 1 axis
Source: Bioengineered. 2021 Dec 21;12(2):12755–66. doi: 10.1080/21655979.2021.1997696 (PMC8809952; doi:10.1080/21655979.2021.1997696)
Supplement: Supplemental Material [file KBIE_A_1997696_SM4107.docx]

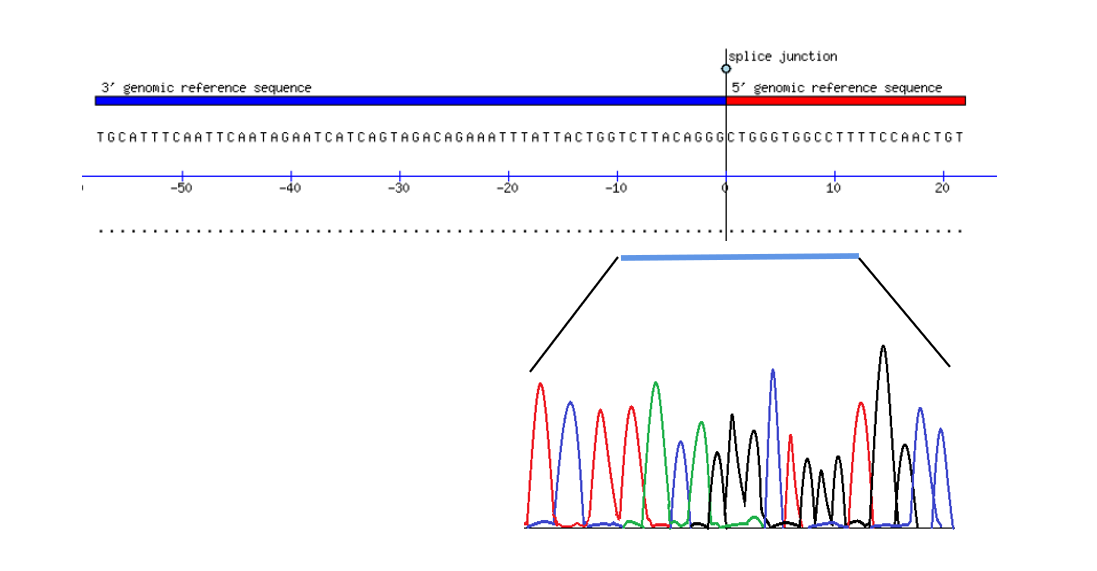


**Supplementary Figure 1. Sanger sequencing was performed to validate the the back splicing of circ_0000423.**


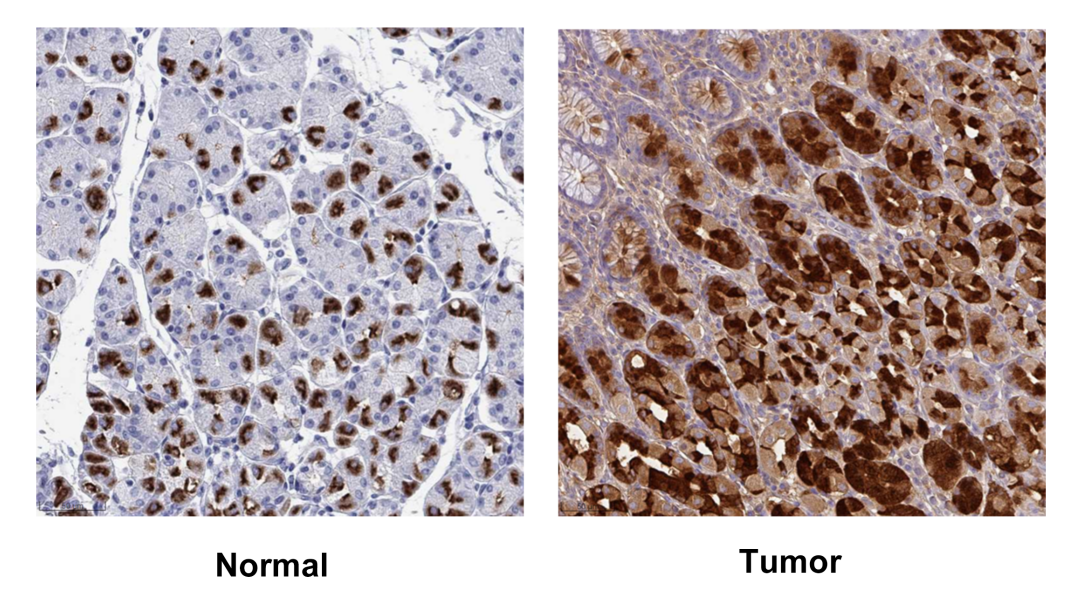


**Supplementary Figure 2. Immunohistochemical method was used to detect the expression of DIXDC1 in a pair of GC tissue / adjacent tissue. Left: adjacent tissues. Right: GC tissues with poor differentiation.**
